# Supplementary material for: Coupling Genome-wide Transcriptomics and Developmental Toxicity Profiles in Zebrafish to Characterize Polycyclic Aromatic Hydrocarbon (PAH) Hazard
Source: Int J Mol Sci. 2019 May 25;20(10):2570. doi: 10.3390/ijms20102570 (PMC6566387; doi:10.3390/ijms20102570)
Supplement: Supplementary file 1 [file ijms-20-02570-s001.zip › Supplementary.pdf]

Supporting Information

Supplementary Tables
Supplement file 1

Table S1 (*tab 1*): List of 123 PAHs, the lowest effect level (LEL) exposure concentrations for each endpoint measured, and the developmental toxicity bins of each PAH.
Table S2 (*tab 2*): List of DEGs (fold-change > 1.5, adjusted p-value < 0.05) for 14 of the 16 PAHs. 3-NF and 1,5- DMN had zero DEGs.
Table S3 (*tab 3*): Top 5000 genes by coefficient of variation (CV) across the 18 treatments (16 PAHs, 2 controls).
Table S4 (*tab 4*): Genes that are common to 1, 2, 3, 4, 5, or 6 of the cluster B PAHs.

Supplement file 2

Table S5: Significant ($p < 0.05$) functional pathways associated with each of the 16 PAHs. Abbreviations: t - Number of genes in the term, Q – number of genes in the query list, Q&T – Number of genes in query list (Q) that are present in T, Q&T/Q – Union of Q and T as a fraction of Q, term ID – gene ontology term ID

Supplementary Figure

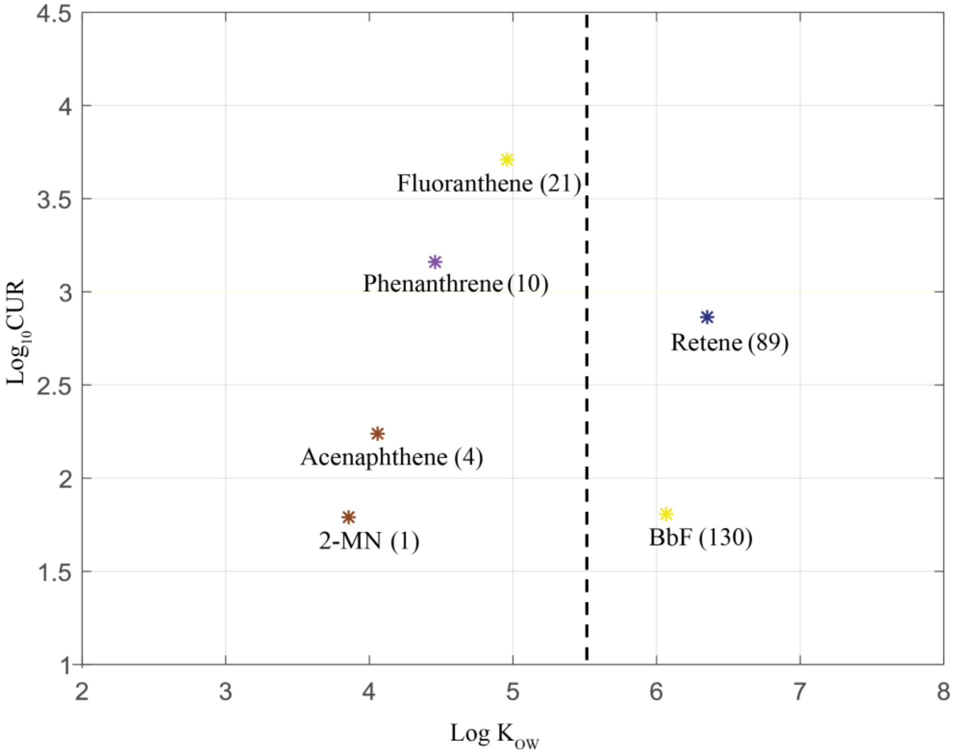

Figure S1: Concentration Uptake Ratio (CUR) for six PAHs compared to log Kow. Zebrafish embryos were exposed to three concentrations (5.39, 11.6, and 25 μM) of the six PAHs anthracene, acenaphthene, phenanthrene, fluoranthene, retene, and BbF, from 6 to 48 hpf. CUR was computed from the ratio of the concentration inside the embryo and to the nominal medium concentration. The color of the data point represents the developmental toxicity bin the PAH clustered into: 2-MN and acenaphthene: bin 6, phenanthrene: bin 7, fluoranthene and BbF: bin 5, and retene: bin 1. The dotted line is at log Kow =5.5. The number of DEGs associated with each PAH are in parentheses.
